# Gut microbiome intervention in gut-brain axis and effects on vascular dementia with prospects on non-pharmacological intervention

**DOI:** 10.1016/j.bbrep.2026.102827

**Published:** 2026-10-02

**Authors:** Yash Maurya, Elizabeth Annie George, Arkadeep Roy, Gayathri J. Menon, K.R. Harshitha, K. Priyadharshini, Sudha Ramaiah, Anand Anbarasu

**Affiliations:** a Department of Biotechnology, School of Biosciences and Technology, Vellore Institute of Technology (VIT), Vellore, Tamil Nadu, 632014, India; b Department of Bio-Sciences, School of Biosciences and Technology, Vellore Institute of Technology (VIT), Vellore, Tamil Nadu, 632014, India

**Keywords:** Ayurveda, Chinese medicines, Gut-brain axis, Gut-microbiome, Vascular dementia

## Abstract

Vascular dementia (VD) is the second leading cause of cognitive impairment after Alzheimer's disease. Current treatments focus primarily on managing vascular risk factors with no effective interventions for repairing existing brain damage. Recent research underscores a critical link between gut microbiota and brain function through immunological, hormonal and metabolic pathways. Alterations in the gut microbiome influence vascular cognitive impairment *via* mechanisms such as dysbiosis, induced intestinal permeability, systemic inflammation, oxidative stress and blood-brain barrier disruption. Microbial metabolites including butyrate promote neuroprotection and maintain vascular integrity, whereas trimethylamine N-oxide is associated with vascular injury and cognitive decline. Restoration of microbial balance through probiotics, plant-based bioactive compounds and traditional medicines has shown potential in preclinical models for regulating inflammation, improving cognitive outcomes though clinical validation is limited. Non-pharmacological approaches like electro-acupuncture further modulate the microbiome and serotonin regulation. This review explores how changes in gut microbiome influence vascular cognitive impairment and discusses therapeutic strategies for restoring microbial balance. Integrating contemporary and traditional strategies, gut microbiome modulation emerges as a potential therapeutic target for the prevention and management of VD although its clinical applicability remains to be fully established.

## Highlights

• Gut dysbiosis contributes to neurovascular and cognitive dysfunction in VD.
• Genetic evidence supports causal microbiome-VD associations.
• Microbiome targeted interventions show neuroprotective efficacy in VD.
• Traditional medicine offers multi-target gut brain axis modulation.

## Graphical abstract

**Figure:**
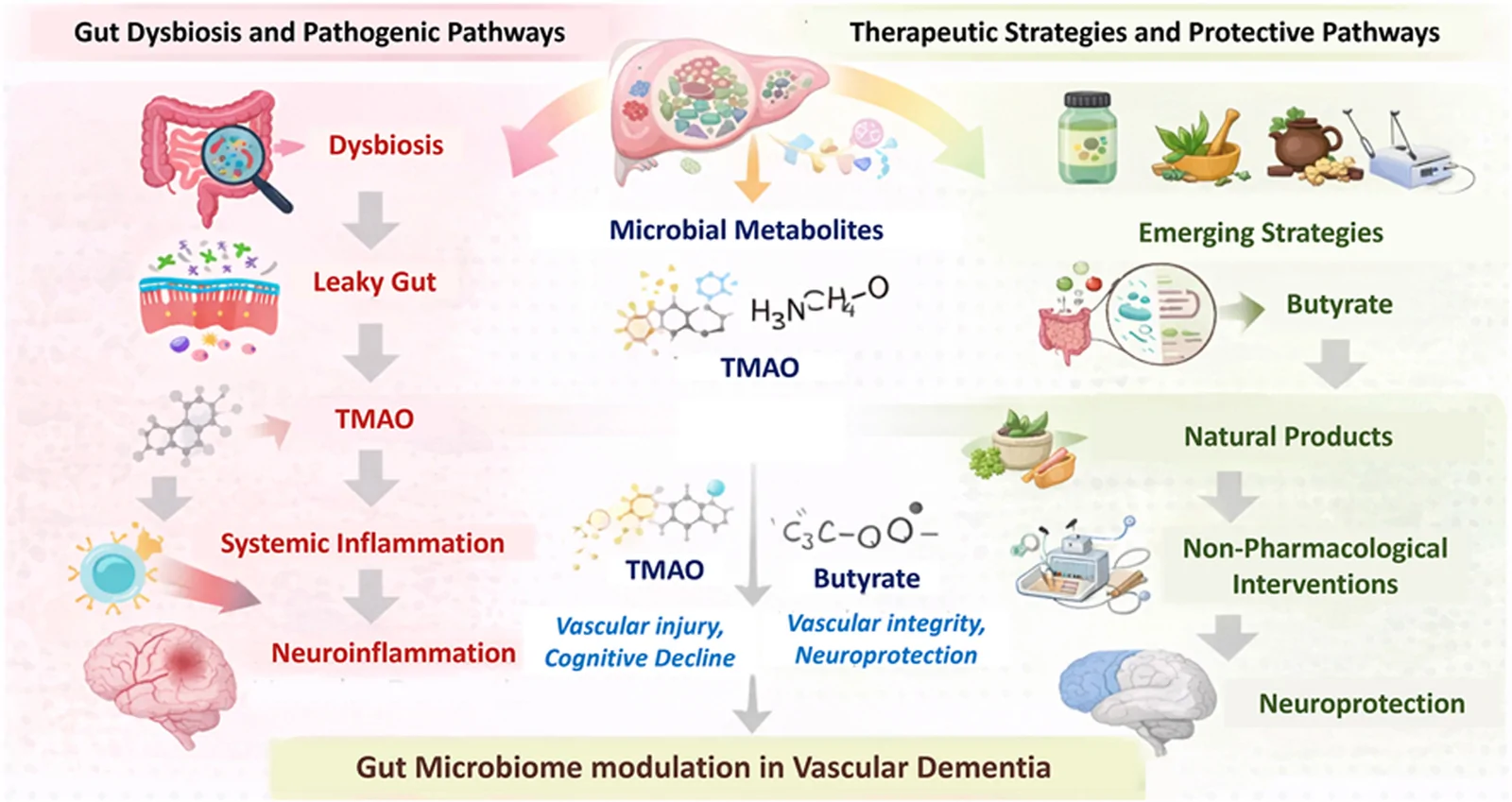

## 1 Introduction

Vascular dementia (VD) develops when the brain's blood vessels become damaged or occluded, leading to reduced oxygen and nutrient supply to nerve cells. Over time, this poor circulation leads to progressive cognitive decline, affecting memory, reasoning, and attention. It is the second most common cause of dementia after Alzheimer's disease and may account for up to one fifth of all dementia cases around the world [1]. The condition can arise suddenly after a major stroke or appear gradually as small vessel disease accumulates unnoticed over the years. Risk factors overlap with many modern health problems such as long-term high blood pressure, diabetes, and lipid disorders and its prevalence continues to rise alongside longer life expectancy. At present, most treatment efforts focus on preventing new strokes or managing heart and metabolic conditions [2]. However, these approaches remain largely preventive and do not adequately address existing neuronal and microvascular damage. This shortfall has motivated researchers to search for new biological pathways that might protect or even restore brain health.

In recent years, attention has shifted toward the gut as an unexpected contributor to brain health. The gut-brain axis describes a constant two-way communication system linking the intestinal microbiota with the central nervous system through neural, immune, and hormonal routes [3]. The vast community of microbes living in the intestine influence emotion, cognition and inflammation through chemical messengers and metabolic products. About ninety percent of the neurotransmitters such as serotonin are produced in the gut highlighting its systemic influence [4]. When this internal ecosystem becomes unbalanced, a state called dysbiosis occurs. The intestinal lining may weaken, allowing bacterial toxins to leak into the bloodstream and trigger immune reactions that travel as far as the brain. These inflammatory molecules can damage the blood-brain barrier (BBB), activate glial cells and set off oxidative stress that worsens vascular injury [5]. In contrast, a balanced microbiome generates protective short-chain fatty acids (SCFAs) such as butyrate, which contributes to host physiological homeostasis [6]. Harmful compounds like trimethylamine N-oxide (TMAO), on the other hand, are associated with endothelial dysfunction and greater risk of cognitive decline [7].Targeting the gut-brain axis is therefore emerges as a promising therapeutic strategy in mitigating vascular damage and cognitive decline in VD.

However, current evidence is largely correlative and the role of microbiome alterations and VD is not fully understood. As evidence grows, scientists are exploring ways to restore microbial balance as a possible therapeutic route. This review synthesizes current evidence from contemporary research and traditional knowledge to examine how the gut microbiome influences the onset and progression of VD. It summarizes the molecular mechanisms linking microbial imbalance to vascular injury and neurodegeneration, and reviews interventions that restore microbial health. Special focus is placed on probiotics, plant-based molecules, and integrative non-pharmacological strategies such as electroacupuncture [8]. Together, these insights point toward a unified view of gut and brain health that may inspire new preventive and therapeutic approaches for cognitive decline.

## 2 Gut Microbiome Alterations in Aging and Cognitive Frailty

The imbalance of gut microbiota is a key mechanism in neurodegenerative diseases and age-related frailty [9]. Dysbiosis promotes systemic inflammation and metabolic imbalance. Animal models show increased amyloid beta and tau-related pathologies associated with Alzheimer's disease [1]. Microbial metabolites play diverse roles in host-microbiome interactions, influencing disease progression [10,11]. Pre-clinical studies using probiotics, prebiotics, and fecal microbiome transplantation have shown decreased neuroinflammation and cognitive deficits though clinical validation is lacking (Fig. 1) [12]. The gut-muscle axis decisively influences cellular senescence and sarcopenia, where pro-inflammatory mediators from dysbiotic microbiota enhance muscle degeneration through chronic low-grade inflammation [13]. Aging related microbiome profiling reveals lower *Firmicutes* and higher *Bacteroidetes*, *Proteobacteria* and *Verrucomicrobiota*, which correlates with higher brain age and cognitive decline with mediation analysis showing brain age partially mediates the microbiome alteration and cognition [14]. However, despite these associations, most of the current evidence is correlative and are largely based on the preclinical models which limits conclusions about causality. The inconsistencies exist across the studies regarding the microbial signatures as well as their functional characteristics likely due to the difference in study populations, methodologies and analytical pipelines. Additionally, whether the microbial alterations are the primary drivers of VD or consequence of disease progression remains unclear. Therefore, although restoration of microbial balance represents a promising therapeutic avenue, its translational potential needs validation through well-designed human intervention studies. Collectively, these findings underscore the relevance of elucidating the underlying core biological mechanisms linking gut dysbiosis to neurovascular dysfunction and cognitive decline in VD.

**Fig. 1:**
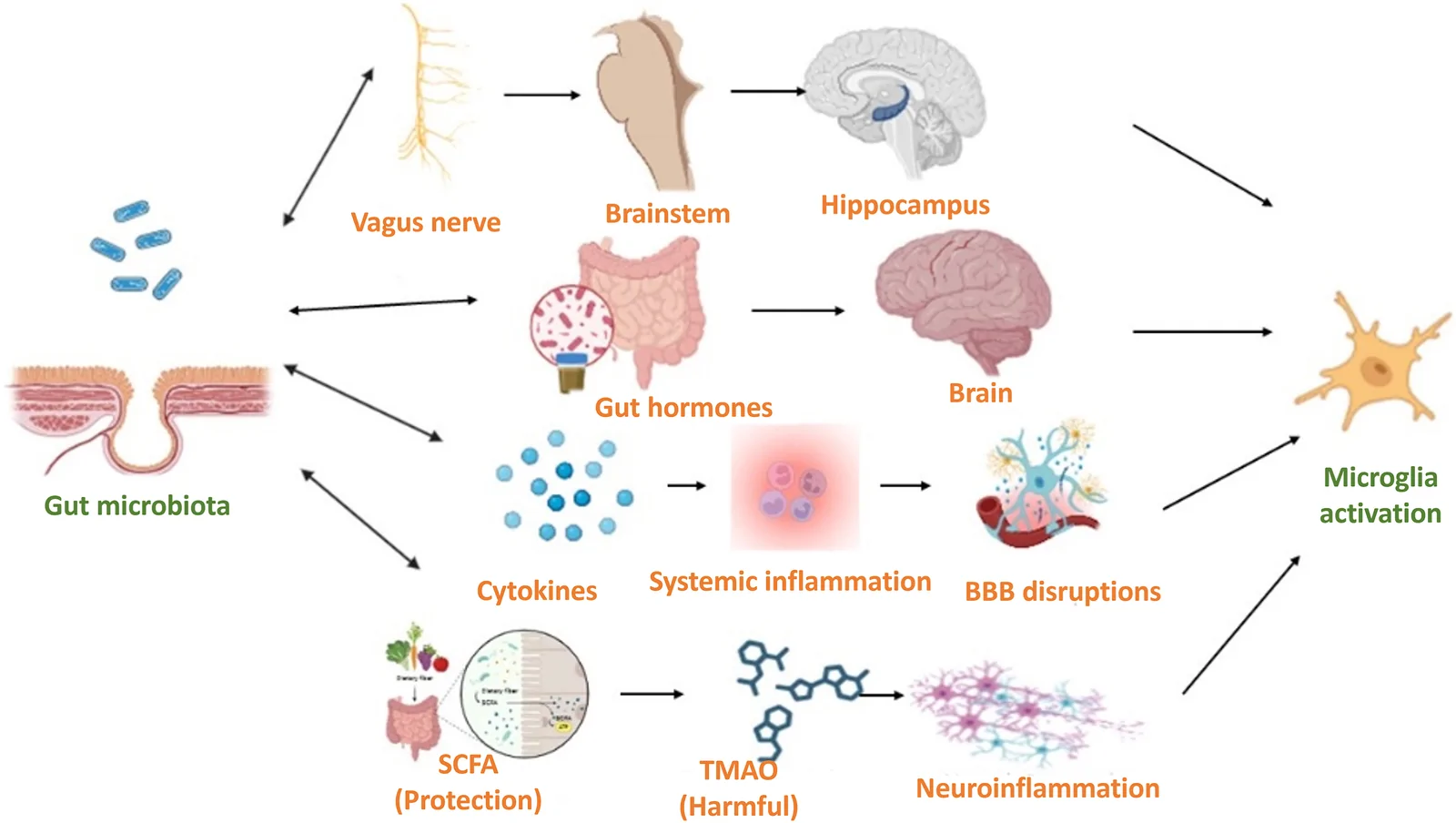
Schematic representation of the core mechanisms of the gut-brain axis. The gut microbiota communicates bidirectionally with the brain through neural, immune, and metabolic pathways. Dysbiosis alters gut barrier integrity and systemic inflammation, promoting BBB disruption, microglial activation, and neuroinflammation that contribute to cognitive decline and vascular dementia.

## 3 Core Mechanisms Underlying the Gut-Brain Axis

The gut-brain axis is a dynamic network of bidirectional communication between the gastrointestinal tract and the central nervous system, mediated through neural, endocrine, immune and metabolic pathways. The gut microbiota can influence brain function through the axis both indirectly and directly [15,16]. Dysbiosis, refers to disruption in composition and function of microbes which may shift host-microbe interactions towards pathological outcomes [17]. It may facilitate systematic dissemination of the microbial components contributing to downstream effects [18]. These may contribute to neuroinflammatory responses and vascular dysfunction [19]. Furthermore, microbial metabolites influence multiple host regulatory pathways. Neural circuits mediating gut-brain signalling provide an anatomical route for microbial signals, while endocrine modulators (e.g., peptides, hormones) regulate gut and brain cross talk. These interconnected networks collectively determines whether the net impact on brain health is protective or detrimental [20]. Within this framework, the microbial metabolites emerge as the key mediators exerting both protective and detrimental effects on neurovascular and cognitive processes.

### 3.1 Protective regulation: SCFAs – butyrate

SCFAs notably acetate, propionate and butyrate are microbial fermentation products of non-digestible dietary fibre, and act as potent modulators of host physiology. Among them butyrate has gained attention for its neuroprotective roles [21]. At the molecular level, butyrate functions as a histone deacetylase (HDAC) inhibitor, facilitating epigenetic activation of neurotrophic genes including brain-derived neurotrophic factor (BDNF) which support synaptic plasticity and neuronal survival (Fig. 2) [22]. In the immune compartment, butyrate influences microglial activation by encouraging anti-inflammatory phenotypes, lowering pro-inflammatory signalling and reducing ROS production. Additionally, by strengthening gut barrier integrity, it limits systemic inflammatory exposure to brain. Translational human studies are more limited, but epidemiological links between high fibre diets, favourable SCFAs profiles, and better cognitive outcomes support its relevance in humans [23]. However, despite the strong mechanistic evidence supporting butyrate's neuroprotective role, its translational challenge persists. Many studies use dietary fibre as an indirect measure of SCFAs production which may not fully reflect the actual butyrate levels. Additionally, the inter-individual variability in the gut microbiome composition can influence the synthesis of butyrate complicating the standardized treatment strategies. A key change remains achieving sustained gut-targeted increase in butyrate producing taxa while accounting in variability in dosage, delivery and microbial ecology. Nevertheless, SCFAs based strategies represent a promising, low risk therapeutic avenue to harness microbiota derived molecular protection for brain health [24]. In contrast to these protective effects certain microbial metabolites exert deleterious effects on neurovascular integrity and cognitive function.

**Fig. 2:**
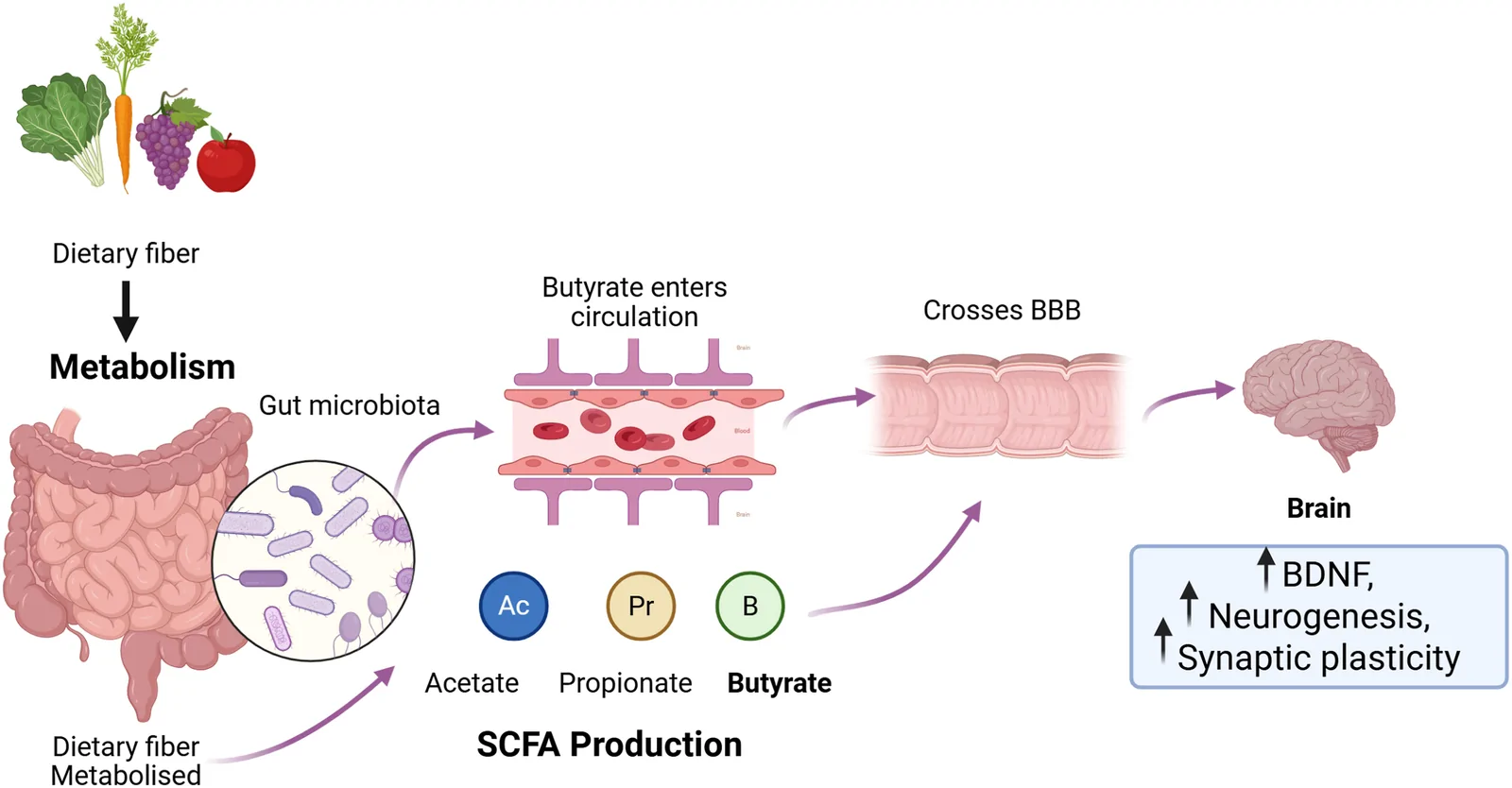
Gut microbiota derived SCFAs and their role in gut brain axis signaling. Dietary fibers metabolized by SCFAs primarily butyrate, it enters circulation crosses BBB and enhances BDNF expression promoting neurogenesis and synaptic plasticity.

### 3.2 Harmful regulation: TMAO

TMAO is a gut microbe-derived metabolite produced when dietary substrates such as choline, l-carnitine or betaine are converted to trimethylamine (TMA) and subsequently oxidized in liver [25]. Mechanistically, TMAO exacerbates oxidative stress by promoting ROS generation, decreasing antioxidant enzyme activity and impairing redox homeostasis in neuronal and glial cells [26]. Simultaneously, TMAO activates pro-inflammatory signals through NF-κB and the NLRP3 inflammasome, leading to elevated cytokine levels and microglial activation [27,28]. TMAO may also induce endothelial dysfunction and further compromise the BBB, thereby facilitating entry of peripheral inflammatory signals [29]. In animal studies, TMAO supplementation impairs learning, memory and synaptic plasticity, whereas interventions reducing TMAO or its microbial production ameliorate cognitive deficits [30]. Epidemiological and analytic human data lend some support as elevated levels of TMAO has been associated with increased odds of cognitive impairment. Although several studies associated with elevated TMAO levels with cognitive impairment, the findings remain inconsistent across large cohort studies. Some studies failed to demonstrate the significant association with incident dementia highlighting the potential factors such as renal function dietary habits and microbiome composition. These discrepancies suggest TMAO as a biomarker of metabolic dysfunction rather than a direct causative agent. Overall, TMAO may function by potentiating oxidative stress, inflammation, vascular or neural impairment [31]. Future research should focus on casual mechanisms and evaluating interventions targeting microbial TMAO synthesis or hepatic oxidation pathways. Beyond this metabolite derived effects, the structural disruption of the gut barrier integrity represents another critical mechanism linking dysbiosis to systemic and neuroinflammation.

### 3.3 The inflammatory pathway: leaky gut and inflamed brain

A cornerstone of gut-brain pathology is so called “leaky gut” phenomenon, in which the intestinal epithelial barrier becomes compromised, allowing bacterial-derived molecules such as LPS and pro-inflammatory cytokines to breach into systemic circulation [32]. These molecules can directly damage endothelial cells of the BBB or trigger cascade effects by activating peripheral immune cells that release further mediators, leading to BBB disruption and increased CNS exposure to inflammatory agents [5]. The compromised BBB then becomes permissive to immune cells and cytokines, initiating microglial activation, astrogliosis, and local cytokine production [33]. Activated microglia adopt a pro-inflammatory phenotype, with upregulation of key inflammatory pathways and reactive oxygen species (ROS) generation, which in turn causes synaptic dysfunction, neuronal apoptosis, and cognitive impairment [34]. Evidence from animal models shows that gut barrier disruption correlated with increased hippocampal inflammation and memory deficits. Additionally, importantly, feedback loops between the CNS and gut may further exacerbate barrier dysfunction creating a feed-forward cycle [35]. Interventions that restore epithelial tight junction integrity or block peripheral to central inflammatory signalling have shown promise in preclinical settings, although clinical validation remains limited [36]. While these pathways provide the biological plausibility, establishing the causal relationships between gut microbiota and VD necessitates robust human evidence derived from genetic and epidemiological approaches.

## 4 Human Genetic Evidence Bridging Correlation and Causation

Genetic epidemiology research has advanced understanding of causal pathways between gut microbiota, their metabolites, neuro-inflammatory mediators, and neurovascular disease, including VD and cerebrovascular disease [37]. Mendelian randomization uses genetic variants as instrumental variables to infer causal associations between exposures and disease outcomes [38]. Genome wide association studies (GWAS) indicate gut bacteria, such as *Allisonella* and *Paraprevotella*, are associated with increased risk of ischemic stroke, whereas others including *Barnesiella* and *Intestinimonas* appear protective. Complementary metabolomic data further suggest that microbial-derived metabolites contribute to cerebrovascular disease [39]. A similar model is observed in neurodegenerative dementias, where some microbiota modulate susceptibility across dementia subtypes.

For instance, several genera and families, including *Desulfovibrionaceae, Lactobacillaceae*, and *Ruminococcus gnavus*, are associated with increased dementia risk whereas *Lachnospiraceae* and *Butyricimonas* exhibit potential neuroprotective effects. These taxa modulate inflammation and amyloid beta processes in the central nervous system, consistent with experimental findings [40]. Additionally, inflammatory cytokines, interleukin 18 and macrophage migration inhibitory factor, have been implicated in VD however mediation analyses suggest that microbial effects may occur independently of systemic inflammation indicating alternative mechanistic pathways [41]. Therapeutically fecal microbiota transplantation has demonstrated potential in preclinical models by reducing amyloid beta deposition, modulating neuroinflammation and restoring BBB integrity although clinical evidences remain limited [42]. While Mendelian randomization strengthens causal inference these findings are still constrained by the inherent methodological assumptions such as pleiotropy and population bias in GWAS datasets. Moreover, the microbial taxa identified may serve as the indirect drivers rather than direct functional drivers, necessitating the need for further experimental validations [43].These casual insights provide a critical foundation in the development of targeted microbiome-based therapeutics aimed in modulating disease associated pathways in VD.

## 5 Emerging Strategies and Preclinical Interventions

### 5.1 Probiotics, prebiotics and postbiotics

According to recent preclinical research, changing the gut microbiota maybe a treatment option for neurodegenerative diseases and cognitive decline through the microbiota gut-brain axis. Evidence shows that probiotic supplements change gut microbial composition, repair the intestinal barrier, affect inflammation and the immune system, and ultimately influence brain function [44]. For instance, the bacterium *Clostridium butyricum*, which produces butyrate, as shown neuroprotective effects in different animal models. One study found that *C. butyricum* improved cognitive dysfunction in mice with Alzheimer's disease induced by ICV-STZ. It restored hippocampal and cortical neurons, reduced phosphorylated tau (pSer404) attenuated TLR4/MYD88/NF-κB signalling and enhanced colonic tight-junction protein expression (Occludin and ZO-1) along the modulation of gut microbiota [45]. Another study involving diet-induced obese mice showed that those supplemented with *C. butyricum* had better cognition and synaptic structure. Their hippocampal gene expression shifted in a way that was opposite to typical Alzheimer's profiles. They also experienced an improved gut barrier with less endotoxemia, under reshaping of a gut microbiota. Faecal microbiota transplantation from *C. butyricum* -treated donors resulted in cognitive benefits [46]. The beneficial effects are likely due to SCFAs particularly butyrate, produced by *C. butyricum*. These substances act on various host cells-including intestinal, liver, immune, and neural cells primarily through SCFA-mediated mechanisms [47]. More generally, shows that probiotics and prebiotics affect the MGB axis through immunological and hormonal signalling, neurotransmitter system, metabolites, and the vagus nerve [48]. Targeted probiotic therapy presents a promising preclinical strategy with limited evidence supporting its clinical efficacy. However, the findings highlight the therapeutic potential of the probiotics but the evidences remain restricted to the animal models. Variation in the host physiology, microbiome complexity and the environmental factors limit the clinical translation. In addition to this the variability in probiotic strains, dosage and duration of the treatment complicates the standardization of therapeutics.

Prebiotics are substrates that specifically utilized by host microorganism conferring a health benefit which mainly include non-digestive carbohydrates such as galactooligosaccharides, chitosan oligosaccharides [49]. These compounds are not digested in the upper gut and reach the colon, where they act as selective substrate for growth of beneficial bacteria. Prebiotics are fermented by gut microbiota to produce SCFAs which contribute to host microbiome interactions. SCFAs enhance expression of tight junction protein ZO-1 and Occludin, activate G-protein coupled receptor such as GPR41/43 and Nrf2/HO-1 antioxidant pathway, which reduce stress and inflammation. Postbiotics are the preparation of inanimate microorganism and their components confer a health benefit on the host [49]. They act through paracrine like signalling. They are important when it is risky to administer live microorganism, as is the case in critically ill or immunocompromised patients. Postbiotics modulates host immune and metabolic responses through microbiota derived bioactive compounds. Beyond microbial supplementations strategies, natural products and multi-target herbal formulations have emerged as complementary approaches capable in modulating the gut microbiota and associated neuroinflammatory pathways in VD.

### 5.2 Natural products and herbal formulations

Alongside single-strain probiotics, various natural bioactive compounds and multi-herbal blends, especially those from traditional medicine, have attracted interest for their ability to modulate gut microbiota and address inflammation/neurodegeneration in the brain [50]. Baicalein, obtained from the *Scutellaria baicalensis* plant, is a significant molecule that shows neuroprotective properties by reducing inflammation in the hippocampus, altering the gut microbiome, and blocking the TLR4/MyD88/NF-κB pathway in microglia. Mechanistic links *via* gut-immune-brain interactions are proposed, although not every study directly assesses alterations in the microbiome [51]. In ischemia-reperfusion injury models, flavonoids such as baicalin improve cognition and modify the gut microbiome. In models of vascular dementia, traditional Chinese medicine (TCM) formulations such as Xinshubao and Tongqiao Yizhi decoction, along with other decoctions, have demonstrated the ability to improve synaptic plasticity, reduce oxidative stress, and restore equilibrium to gut microbial profiles [52]. Despite the existing absence of direct clinical evidence, the comprehensive multi-target nature of the herbal formulation aligns with the concept of gut-brain axis modifications, simultaneous impacts on microbial diversity, gut integrity, immune/inflammatory communication, and CNS robustness. Alongside proposed reductions in circulating pro-inflammatory cytokines and expected effects on networks and pharmacology through gut/brain-axis pathways, a recent observational study that integrated herbal medicine and electro acupuncture indicated that patients with mild cognitive impairment showed enhancements in their cognitive abilities [51]. Consequently, the integrative capacity of natural products suggests a potential link between microbiome research and neurodegenerative treatments, although direct clinical evidence remains limited [53]. Nevertheless, the mechanistic understanding remains limited as the studies do not directly link the microbiome modulation and the cognitive outcome. The complexity of multi component herbal formulations limits the reproducibility and most evidence is still preclinical with limited clinical validation. Complementary to these bioactive compounds, non-pharmacological interventions have also emerged as potential modulators of gut-brain axis through integrated neural, immune and microbial pathways.

### 5.3 Non-pharmacological interventions

Therapies using non-pharmacological neuromodulators, which are gaining recognition, can influence the gut-brain axis. Electroacupuncture has been shown in preclinical studies to influence immune, gut and neural pathways simultaneously [54]. For instance, electroacupuncture lowered colonic 5-HT and hippocampal JNK signalling, diminished serum hippocampal levels of IL-1, IL-6, and TNF-, enhanced cognitive abilities, and changed the composition of gut microbes in rodent Alzheimer's disease models by promoting beneficial genera such as *Lactobacillus* and *Bifidobacterium* [8]. Another study utilizing multi-omics integration revealed that the gut microbiota is crucial for electroacupuncture. Mice that received antibiotics, leading to a reduction in their microbiota, demonstrated that the gut microbiome is crucial in the electroacupuncture-cognition pathway [55]. The fundamental process is becoming evident. Immune signaling, metabolite production, microbial and environmental influences, secretion, motility, and the central nervous system circuitry linked to memory and cognition stimulate vascular and splanchnic peripheral nerves. A recent review of TCM methods for vascular cognitive impairment identified the modification of gut microbiota through acupuncture, moxibustion, and herbal treatments as a key mechanism [56]. This non-drug approach is especially appealing for integrative medicine methods, although clinical application, protocol and understanding the underlying mechanisms still require enhancement [57]. However, despite of promising results the clinical use of acupuncture remains uncertain due to the limited human studies and lack of standardized protocols. Variability in the treatment parameters including acupoint selection stimulation intensity, duration complicates the clinical translation. A comprehensive summary of the therapeutic strategies, their microbial targets, mechanisms and supporting clinical and preclinical evidence is presented in Table 1. Despite promising preclinical findings, the overall evidence remains heterogenous with varied outcomes across the studies. Importantly host specific factors such as diet, age, microbial composition significantly influences the therapeutic responses thereby limiting the clinical translation. Building on these emerging non-pharmacological approaches, traditional therapeutic systems offer an integrative strategy combining microbiome modulation with multitarget mechanisms to address VD.

**Table 1:** Summary of emerging microbiome based therapeutic strategies.

Emerging Strategies	Metabolites	Microbial target/Mechanism of action	Limitations	Evidence *(Nature)*	References
**Probiotics** (*Clostridium butyricum)*	SCFAs (Butyrate)	Reported to enhance SCFAs-producing bacteria and gut barrier integrity; inconsistent outcomes	Strain specific effects; small sample sizes; lack of human validation; influenced by diet, age, microbiota	Animal studies *(Causal)*	[45]
**Prebiotics** (Galactooligosaccharides)	SCFAs (Butyrate, acetate)	Associated with improving beneficial microbiota and gut integrity; population-dependent effects	High inter-individual variability; diet- and microbiota-dependent	Preclinical *(Associative)*	[49]
**Postbiotics** (Microbial metabolites)	SCFAs, bioactive peptides	Modulates immune response; reduces inflammasome activation	Dose variability; limited *in vivo* validation	Preclinical *(Mechanistic)*	[49]
**Natural Compounds** (Baicalein, curcumin, ginsenosides)	SCFAs, amino acid metabolites	Reported to reduce oxidative stress and neuroinflammation; inconsistent translation	Lack of clinical trials; poor bioavailability; host-dependent metabolism	Animal studies *(Mechanistic)*	[50]
**TCM** (SMYZG)	Multi-metabolites	Associated with microbial balance restoration; anti-inflammatory; model-dependent evidence	Lack of standardization; multi-component variability	Animal studies *(Associative)*	[52]
**Ayurvedic Interventions** (Ashwagandha)	Neuroactive compounds	Reported to modulate microbiota and neuroinflammation; limited mechanistic clarity	Limited clinical validation; variability in formulations; affected by comorbidities or age	Preclinical *(Associative)*	[51]
**Non-Pharmacological** (Electroacupuncture)	SCFAs, neurotransmitters	Modulates gut–brain signalling; heterogeneous outcomes	Variability in protocols; smaller clinical datasets	Animal + emerging clinical *(Associative)*	[8]

## 6 Traditional intervention for VD

### 6.1 Chinese medicine

The investigation of natural products and herbal formulations is increasingly promising, primarily because they can simultaneously target multiple components of the gut-brain axis. These interventions have been reported to modulate the neuroinflammation, cerebrovascular dysfunction and the gut microbiota dysbiosis primarily in the preclinical studies. This holistic, multi-target approach is a cornerstone of TCM, which aligns with modern systems-level views of the gut-brain connection. This section reviews evidence for TCM's complex formulations, now being studied for their capacity to modulate the gut microbiome, reduce neuro-inflammation, and enhance cognitive function specifically in VD models. Table 2 summarizes different Chinese medicines showing effects on gut-brain axis, neuro-inflammation and their mode of actions. Most findings are derived from the preclinical studies further clinical validation in VD is essential. Within this comprehensive frame work TCM offers a multi target therapeutic approach that aligns with gut-brain modulation through its combined effect on the microbial composition, neuroinflammation and vascular function in VD.

**Table 2:** Neuroprotective and gut-brain axis modulating herbal compounds and formulations in cognitive enhancement.

Herb	Composition	Key Components	Mechanism	References
Baicalein	*Scutellaria baicalensis*	Flavonoids	Modulates gut microbiota; reduces neuroinflammation	[58]
Curcumin	*Curcuma longa*	Phenols	Modulates microbiota; reduces oxidative stress markers; improves memory function	[57]
Tongnao Yizhi granules	Compound formula	Multi-herb	Alters gut microbiota; improves learning and memory	[57]
Wuzang Wenyang Huayu decoction	Compound formula	Multi-herb	Enhances neurogenesis; promotes autophagy; modulates metabolites	[59]
Dingzhi Yicong formula	Compound formula	Multi-herb	Improves cognition; restores microbial balance	[59]
Shibing Xingnao granules	Compound formula	Multi-herb	Improves cognitive function; neuroprotective effects; modulates gut microbiota	[59]
Schisandrin	*Schisandra chinensis*	Lignan	Improves intestinal barrier integrity; reduces endotoxin leakage	[60]
Panax Notoginseng saponins	*Panax notoginseng*	Saponins	Modulates microbiota; protects against ischemic injury	[61]
Huanglian Jiedu decoction	Compound formula	Multi-herb	Anti-inflammatory; reduces tau pathology	[62]
*Gastrodia elata*	*Gastrodia elata*	Natural compounds	Modulates neurotransmitters; neuroprotective	[63]
*Cistanche deserticola*	*Cistanche deserticola*	Polysaccharides	Antioxidant; reduces oxidative stress	[64]
Huayu Ditang Yizhi Formula	Compound formula	Multi-herb	Improves cognition; increases microbial diversity	[27]
Epimedium flavonoids	*Epimedium*	Flavonoids	Enhances neurotransmitters; improves cognition	[65]
Ginsenoside Rd	*Panax ginseng* or *Panax notoginseng*	Saponin	Promotes autophagy; anti-inflammatory	[59]
Ginsenoside CK	*Panax ginseng* or *Panax notoginseng*	Saponin	Enhances autophagy; improves learning and memory	[59]
Astragaloside IV	*Astragalus membranaceus*	Saponin	Anti-inflammatory; improves cognition	[59]
Lobetyolin	*Codonopsis pilosula*	Alkaloid	Repairs gastrointestinal mucosa; metabolic regulation	[59]
Tetrahydroberberine	*Coptis chinensis*	Alkaloid	Neuroprotective; modulates gut-brain axis	[59]
Saikosaponin E	*Bupleurum chinense*	Saponin	Neuroprotective; anti-inflammatory	[59]
Linoleic acid	Natural plant oils	Polyunsaturated fatty acid	Modulates microbiota; inhibits AChE,	[59]
*Ginkgo biloba*	Leaf extract	Flavonoid/terpene	Improves blood flow; Reduces tau hyperphosphorylation	[66]
Huperzine	*Huperzia serrata*	Alkaloid	Neuroprotective; inhibits AChE	[67]

#### 6.1.1 Baicalein

Baicalein is a flavonoid from *Scutellaria baicalensis*, that demonstrates potent anti-inflammatory and neuroprotective properties in animal models of VD. It has been shown to improve neuroinflammation in hippocampus [57]. It inhibited the activation of the TLR4/MyD88/NF-ΚB signalling pathway. In rats with VD, it was found to be effective in improving the conditions by attenuating hippocampal inflammatory response by the inhibition of the said pathway. Simultaneously, baicalein acts in the gut to modulate the diversity and composition of the intestinal microbiota including the suppression of microbiota associated with inflammation contributing to gut-brain axis regulation in VD [68].

#### 6.1.2 Shen Ma Yi Zhi granule (SMYZG)

SMYZG is a TCM compound composed of four herbs *Panax Ginseng, Gastrodia elata*, *Euonymus alatus*, and *Ligusticum chuanxiong* [69]. In animal models of VD, SMYZG was reported to improve learning and memory, protect the structure of the hippocampus, and suppress inflammatory and oxidative stress responses. It works through multiple signalling pathways, including the Nrf2/HO-1 pathway (antioxidant and anti-inflammatory effects) and the VEGF/Flk-1/p38 MAPK pathway (improving synaptic plasticity) which are closely associated with VD related neuroinflammation and impairment of cerebrovascular system [69].

#### 6.1.3 Ginkgo biloba

*Ginkgo biloba* is one of the most widely studied natural products for cognitive health and has shown promise for both Alzheimer's disease and vascular dementia. It has shown enhance cognitive function in VD, reduce anxiety in dementia, and provide neuroprotection *via* mechanisms including the improvement of brain perfusion (blood flow), antagonization of the platelet-activating factor and alleviating neuroinflammatory injury. These vascular and anti-inflammatory effects are associated to VD pathophysiology and may be partly mediated through gut microbiome interactions [70].

#### 6.1.4 Curcumin

In VD models, curcumin has been shown to improve memory function using mechanisms which reduce the oxidative stress markers and modulating the gut microbiota. Specifically, it was shown to significantly increase the abundance of the bacteria *Robinsoniella.* Such microbial alterations may contribute to gut microbiota mediated neuroprotection, reduced neuroinflammation and improved gut-brain signalling in VD [71].

#### 6.1.5 Wuzang Wenyang Huayu decoction

In a rat model of vascular dementia, this decoction was found to improve learning and memory. Mechanistic studies showed it regulated cellular autophagy in the brain and modulated faecal metabolites related to vitamin B, purine, and pyrimidine metabolism highlighting its role in gut-brain axis regulation in VD [68].

#### 6.1.6 Tongnao Yizhi Granules

Tongnao Yizhi Granules (TYZG), have been identified as having neuroprotective properties in the case of VD [52]. In conjunction with faecal microbiota transplantation, TYZG greatly changed the composition of gut microbiota in VD rats by increasing the Bacteroides/Firmicutes ratio and boosting the presence of good taxa like Actinomycetes. These changes in microbiota were linked to reduced neuroinflammation and improved hippocampal structure consequently, led to better spatial learning and memory performance. The results indicate that the action of TYZG is partly through the modulation of gut–brain microbial interactions, thus presenting a promising microbiota-targeted strategy for cognitive recovery in VD [57].

#### 6.1.7 *Panax notoginseng* Saponins (PNS)

PNS was shown to have a positive impact on flora by increasing the count of *Lactobacillus reuteri*, promoted the production of histidine, and reduced the neuronal injury caused by ischemia. Besides, notoginsenoside R1 (NG-R1), the most important of PNS active ingredients, was found to relieve neuroinflammation by inhibiting astrocyte and microglia activation through the TLR4/MyD88/NF-ΚB signalling pathway inhibition in both the ischemic brain and colon [72]. This mechanism led to improved BBB function, less cytokine secretion, and more equal distribution of gut bacteria, which all together provided more neurovascular protection and facilitated cognitive and functional recovery after ischaemia-induced injury. Similarly, Ayurvedic systems provide complementary holistic approaches that integrate gut health, systemic metabolism and neurocognitive function.

### 6.2 Ayurvedic approaches

Ayurveda provides a holistic framework linking gut health and neurological functions. The ayurvedic concepts presented as hypothesis generated frameworks ad should be interpreted with the emerging scientific evidence rather than the direct mechanistic equivalence. Table 3 summarize the ayurvedic herbs showing effects associated with VD and neuroinflammatory pathways. Ayurvedic concepts such as Agni (digestive function) and Ama (metabolic) can be conceptually aligned with gut microbiota balance and systemic inflammation which are implicated in VD although these associations remain inferential. Herbs like Ashwagandha (*Withania somnifera*) an adaptogenic herb, eases stress-induced cognitive impairment by modulation of HPA axis by reduction of corticosterone, balancing dopamine and serotonin and reducing oxidative stress *via* modulating gut microbiota and gut-brain axis signalling [73]. Its bioactive compound Withaferin A reduces the neuroinflammation by inhibition of Interluekins-1β (IL-1β) and enhance autophagy in microglia suggesting the involvement of gut-brain axis in reducing neuroinflammation in VD. Bramhi (*Bacopa monnier*i) another common ayurvedic herb has shown to neuroprotective activity in preclinical studies by its anti-inflammatory and antioxidant activity by inhibiting production of pro-inflammatory cytokines [74]. Triphala, has been reported to improve the cognitive function and neuroprotection in the hippocampus by the activation of Nrf2/HO-1 axis reducing oxidative stress and inflammation with possible effects on gut microbiota composition and the production of metabolites [75]. Certain interventions may influence the gut-brain axis dynamics however their role in VD needs further clinical validations. While several mechanisms were supported by preclinical studies, their clinical relevance in VD remains to be fully established. The emerging evidences indicates that VD is not solely a consequence of by cerebrovascular dysfunction but also by the gut microbiota dysbiosis and systemic inflammation. Microbial metabolites such as TMAO contribute to vascular dysfunction and neuroinflammatory damage whereas metabolites like SCFAs support gut barrier integrity and promote neurotrophic signalling. Collectively, these insights provide a mechanistic basis in integrating microbiome targeted traditional interventions into VD management. Despite these promising approaches, significant challenges remain in translating microbiome targeted interventions into standardized and clinically validated therapies for VD.

**Table 3:** Ayurvedic compounds targeting neuroprotection, cognitive enhancement, and gut–brain axis modulation.

Ayurvedic herbs	Mechanism/Pathway	Effect Summary	Classification	Reference
*Withania somnifera* (Ashwagandha)	Modulates neurotransmitters systems; inhibits AChE; reduces HPA axis activity; decreases Aβ aggregation	Enhances memory, executive function, and attention; reduces anxiety and stress; provides neuroprotection	Adaptogen, Neuroprotective, Cognitive enhancer	[76]
Withaferin A (Ashwagandha)	Promotes microglial autophagy (↑LC3B, ↓SQSTM1); inhibits NF-κB/IL-1β signalling	Mitigates neuroinflammation; Enhances cognitive performance	Steroidal lactone, Neuroprotective, Anti-inflammatory	[77]
*Bacopa monnieri* (Brahmi)	Antioxidant (↑SOD, CAT, GPx); inhibits AChE; activates CREB and serotonergic (5-HT) signalling	Improves memory, learning, and attention; reduces oxidative stress and inflammation;	Nootropic, Brain tonic	[78]
Triphala	Activates Nrf2/HO-1 pathway	Ameliorates cognitive deficits	Nutraceutical, Adaptogen	[79]
*Centella asiatica* (Mandukaparni)	Exerts antioxidant, anti-inflammatory and neurotrophic effects	Enhances memory and reduces stress	Cognitive enhancer	[80]
*Tinospora cordifolia* (Guduchi)	Modulates synaptic plasticity markers; boosts antioxidant enzymes	Improves cognitive performance; reduces anxiety-like behaviour	Adaptogen	[81]
*Mucuna pruriens* (Kapikachhu)	Contains natural l-Dopa; inhibits AChE	Neuroprotective and anti-parkinsonian;	Neuroprotective, Anti-parkinsonian	[82]
*Terminalia arjuna* (Arjuna)	Attenuates BBB damage; reduces MMP-2/9	Provides neuroprotection; cardioprotective	Cardioprotective, Neuroprotective	[83]
*Celastrus paniculatus* (Jyotishmati)	Inhibits AChE; reduces neuroinflammation	Improves memory retention.	Nootropic, Cognitive enhancer	[80]
*Acorus calamus* (Vacha)	Prevents mitochondrial damage; inhibits AChE; exerts anti-inflammatory and antioxidant effects.	Reduces memory deficits through neuroprotective mechanisms.	Nootropic, Neuroprotective	[80]
*Panax ginseng* (Asian Ginseng)	Reduces neuronal apoptosis; promotes neurogenesis and synaptogenesis	Improves cognitive performance	Adaptogen, Cognitive enhancer	[84]

## 7 Translational Challenges and Clinical Constraints

Despite significant advances in understanding the role of gut-brain axis in VD, several barriers continue to hinder its clinical translation. The primary limitation is the lack of human evidence as majority of the current findings are derived from the preclinical models while existing clinical studies often lack adequate sample size methodological consistency and validation [85]. The inter individual variation in the gut microbiome composition further complicates the clinical translation. Different factors such as diet, genetics, environmental exposure and disease state also contributes to substantial heterogeneity in microbial profiles thereby limiting the identification of potential biomarkers and reducing the predictability of responses towards therapeutics across populations [86]. The lack of standardization across therapeutic interventions remains a critical constraint. Bioactive peptides, offering specificity in in modulating microbial composition and inflammation, however their clinical translation is limited due to its stability, delivery, potential immunogenicity and insufficient human validation [87,88]. Variability in microbial strains, formulations and integrative interventions limits the reproducibility and comparative studies. The safety and long-term stability of the microbiome modulation is insufficiently characterized [89]. Altering the microbial ecosystems may lead to the unintended consequences including dysbiosis, immune balance underscoring the need for extensive safety assessments. Furthermore, many reports are largely correlative with limited evidence to establish significant relationship with microbial factors and disease progression. Addressing these challenges through clinical validations and standardization of methodologies is necessary for advancing its clinical applicability [90].

## 8 Conclusion and Future Prospects

Gut-brain axis is a promising target for understanding and managing VD. Harmful metabolites like TMAO exacerbates the vascular damage and cognitive decline, where as beneficial microbial metabolites including SCFAs exerts neuroprotection by enhancing the BBB, promoting neurotrophic signalling and reducing oxidative stress. Restoring the microbial balance through probiotics, natural bioactive compounds and traditional Ayurvedic or Chinese medicine formulations reported to have potential, largely based on the preclinical evidence in modulating the metabolic and neuroinflammatory pathways. The non-pharmacological interventions such as electroacupuncture can further enhance the gut microbiota and serotonergic signalling. The future research should focus on elucidating mechanistic studies on pathways and receptors through which the microbial metabolites influence cerebrovascular and neuronal health. Additionally, efforts should also be directed towards standardizing, validating traditional medicine and personalizing microbial therapies by optimizing probiotics, prebiotics and postbiotics by identifying active components and confirming their safety and efficacy in large scale trails. Targeting pathogenic pathways represents another important area of investigation. Finally performing longitudinal human studies are essential to track gut microbiome composition, metabolite profiles, inflammatory markers and cognitive outcomes to identify reliable biomarkers for early detection and intervention. Integrating modern microbiome with traditional medicine could provide multi targeted preventive and personalized strategies to maintain the vascular integrity and cognitive function in the aging populations.

## CRediT authorship contribution statement

**Yash Maurya:** Writing – original draft. **Elizabeth Annie George:** Writing – review & editing, Data curation. **Arkadeep Roy:** Writing – original draft, Conceptualization. **Gayathri J. Menon:** Writing – original draft, Conceptualization. **K.R. Harshitha:** Writing – original draft, Conceptualization. **K. Priyadharshini:** Writing – original draft, Conceptualization. **Sudha Ramaiah:** Writing – review & editing, Project administration, Funding acquisition. **Anand Anbarasu:** Writing – original draft, Supervision, Project administration, Conceptualization.

## Ethics approval and consent to participate

Not applicable.

## Funding Statement

The authors would like to thank the Indian Council of Medical Research (ICMR), New Delhi for funding the research grant IRIS ID: 2021-11889.

## Declaration of Competing Interest

The authors declare that they have no known competing financial interests or personal relationships that could have appeared to influence the work reported in this paper.

## Data Availability

All data generated or analysed during this study are included in this article.
